# Structure of the glucosyltransferase domain of TcdA in complex with RhoA provides insights into substrate recognition

**DOI:** 10.1038/s41598-022-12909-8

**Published:** 2022-05-30

**Authors:** Baohua Chen, Zheng Liu, Kay Perry, Rongsheng Jin

**Affiliations:** 1 Department of Physiology and Biophysics, School of Medicine, University of California, Irvine, CA 92697 USA; 2 NE-CAT and Department of Chemistry and Chemical Biology, Argonne National Laboratory, Cornell University, Argonne, IL 60439 USA

**Keywords:** Structural biology, X-ray crystallography, Clostridium difficile

## Abstract

*Clostridioides difficile* is one of the most common causes of antibiotic-associated diarrhea in developed countries. As key virulence factors of *C. difficile*, toxin A (TcdA) and toxin B (TcdB) act by glucosylating and inactivating Rho and Ras family small GTPases in host cells, which leads to actin cytoskeleton disruption, cell rounding, and ultimately cell death. Here we present the co-crystal structure of the glucosyltransferase domain (GTD) of TcdA in complex with its substrate human RhoA at 2.60-angstrom resolution. This structure reveals that TcdA GTD grips RhoA mainly through its switch I and switch II regions, which is complemented by interactions involving RhoA’s pre-switch I region. Comprehensive structural comparisons between the TcdA GTD–RhoA complex and the structures of TcdB GTD in complex with Cdc42 and R-Ras reveal both the conserved and divergent features of these two toxins in terms of substrate recognition. Taken together, these findings establish the structural basis for TcdA recognition of small GTPases and advance our understanding of the substrates selectivity of large clostridial toxins.

## Introduction

Toxin A (TcdA) and toxin B (TcdB) are two exotoxins produced by *Clostridioides difficile* (*C. difficile*), which are the main causes of *C. difficile* infection (CDI) with variable clinical features including life-threatening pseudomembranous colitis^1–4^. TcdA and TcdB have modular architectures consisting of four major domains: an N-terminal glucosyltransferase domain (GTD), a cysteine protease domain (CPD), a delivery and receptor binding domain (DRBD), and a combined repetitive oligopeptides (CROPs) domain. TcdA and TcdB recognize and enter host cells via receptor-mediated endocytosis^4–9^. These toxins then transport the GTD and the CPD into the cytosol^10–13^, where the CPD cleaves off and release the GTD in the presence of cytosolic inositol hexakisphosphate (InsP6)^14,15^. Once in the cytosol, the GTD inactivates the Rho/Ras-family of small guanosine triphosphatases (GTPases) via glucosylation, leading to disruption of the actin cytoskeleton in target cells and damage of the barrier function of epithelium in the intestine^16–20^.

TcdA and TcdB belong to the large clostridial glucosylating toxin (LCGT) family, which also include *Paeniclostridium sordellii* toxins TcsL and TcsH, *Clostridium novyi* toxin TcnA, and *Clostridium perfringens* toxin TpeL^4,21^. These toxins and many virulence factors from other pathogenic bacteria act by covalently modifying and thus interfering with the physiological functions of small GTPases in host cells, which are essential molecular switches involving in diverse signal transduction pathways^22,23^. TcdA, TcdB, and other members in the LCGT family glucosylate Rho and/or Ras GTPases at the highly conserved threonine residue (for example T35 in Rac1 and Cdc42, T37 in RhoA) using uridine diphosphate-glucose (UDP-glucose) as the sugar donor^18,19,22^. Glucosylation prevents these GTPases from binding to their downstream effector proteins and therefore abolish their functions in many crucial signaling pathways related to morphogenesis, polarity, movement, and cell division^24^.

The sequence identity between the GTD of TcdA and TcdB is only ~ 51%, but both could target the Rho family GTPases (RhoA, Rac1, and Cdc42) with comparable activities, except that GTD^TcdA^ is more efficient at modifying RhoA whereas GTD^TcdB^ is faster at modifying Rac1 based on in vitro time course experiments^18,19,25^. Besides Rho proteins, GTD^TcdA^ could target other GTPases, such as H/N/K-Ras, as minor substrates^26–28^. In a recent study, we demonstrated the structural mechanism by which GTD^TcdB^ recognizes Rho and R-Ras^20^. Here we report the co-crystal structure of GTD^TcdA^ from strain VPI10463 in complex with human RhoA in the presence of UDP-glucose, GDP, Mn^2+^ and Mg^2+^. Comprehensive structural comparisons between the GTD^TcdA^–GTPase complex and the GTD^TcdB^–Cdc42/GTD^TcdB^–R-Ras complexes reveal both the conserved and divergent features of TcdA and TcdB in terms of their substrate selectivity in host cells. These findings advance our understanding of the glucosyltransferase activities of TcdA and TcdB and pave the structural basis for inhibitor design against the GTD.

## Results

### Structure determination and overall structure of the TcdA GTD-RhoA complex

**Figure 1:**
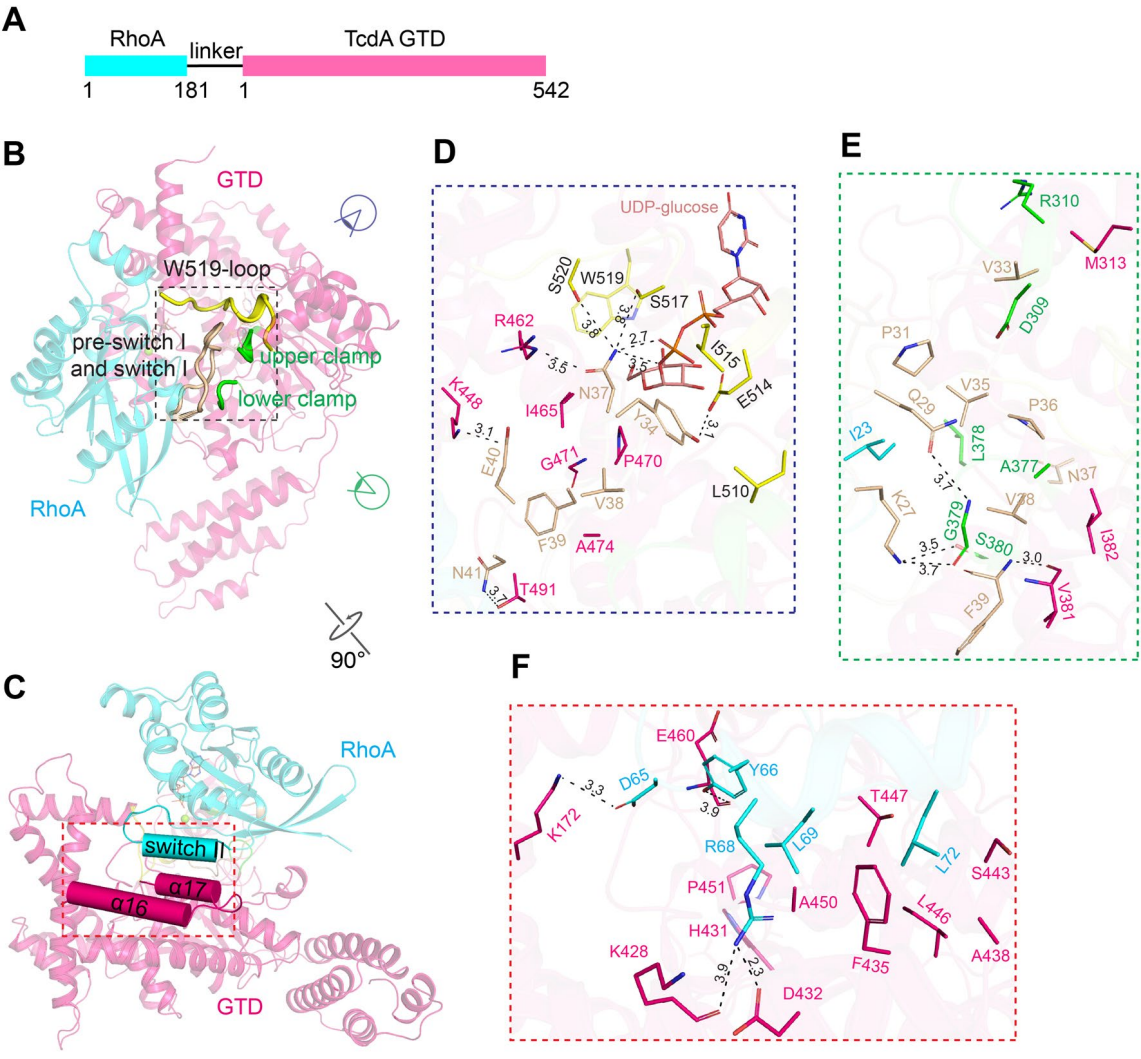
The overall structure of the GTD^TcdA^–RhoA complex. (**A**) A schematic diagram showing the design of the GTD^TcdA^–RhoA chimera protein. (**B**/**C**) Cartoon representations of the GTD^TcdA^–RhoA complex in two different views. GTD^TcdA^ and RhoA are colored hot pink and cyan, respectively. The W519-loop of GTD^TcdA^ is colored yellow, the lower and upper clamps of GTD^TcdA^ are colored green, the pre-switch I and switch I of RhoA are colored wheat. The α16/17 of GTD^TcdA^ and switch II of RhoA are showed as cylinders in (**C**). (**D**/**E**) Close-up views into the interface between GTD^TcdA^ and RhoA focusing on the pre-switch I (**E**) and switch I (**D**/**E**) from two different viewing angles as indicated by purple and green symbols. The interacting residues are colored using the same scheme as that in (**B**). (**F**) Close-up view into the switch II-binding interface, and the interacting residues are colored the same as (**C**).

Like most other enzymes, the GTD transiently binds to and modifies its substrates, and then releases it before engaging the next substrate. This has posed a great challenge for us to prepare stable GTD–substrate complexes for crystallization. To overcome this obstacle, we have developed a strategy to “freeze” the transient interactions between the GTD and its substrates by protein engineering. As reported in one of our recent studies, we designed a fusion protein where Cdc42 or R-Ras was covalently linked to the N-terminus of the GTD of TcdB via a flexible peptide linker^20^. The peptide linker does not restrict interactions between GTD^TcdB^ and its substrates, while the covalent linking increases their local concentrations and thus strengthens the protein–protein interactions^29,30^. At the same time, we mutated the key threonine residue on Rho/Ras (e.g., T35 in Rac1 and Cdc42, T37 in RhoA), the glucosylation target, into an asparagine to prevent the completion of glucosylation in order to stabilize the complex^18^. In this study, we used the same strategy to design a tandem RhoA (residues 1–181)–GTD^TcdA^ (residues 1–542, strain VPI10463) fusion protein, where the two proteins are linked via an 18-amino acid peptide linker (GGGGSGGGSGTGSGGGGS) (Fig. 1A). RhoA carries the T37N mutation to prevent glucosylation. We also introduced a K190A mutation on GTD^TcdA^ to minimize non-specific degradation at this site during protein expression and purification. This mutation is unlikely to affect the activity of GTD^TcdA^, because it is located on the surface of GTD^TcdA^ that is far away from the substrate-binding interface and the UDP-glucose-binding pocket.

The best crystals of the GTD^TcdA^–RhoA complex were obtained in the presence of Mn^2+^ and UDP-glucose that bind to the GTD and Mg^2+^ and GDP that bind to RhoA, and the structure was determined at 2.60 Å resolution (Table S1). The crystals belong to space group C222_1_, and there is one pair of the GTD^TcdA^–RhoA complex in an asymmetric unit with a total buried molecular interface of ~ 1434 Å^2^^31^. The co-factors, Mn^2+^, Mg^2+^, GDP, and the UDP moiety of UDP-glucose, have well-defined electron densities, but the glucosyl moiety of UDP-glucose has weaker density that is likely due to partial cleavage of UDP-glucose during crystallization. The flexible 18-amino acid peptide linker has no visible electron density, implying a highly flexible conformation that would not constrain GTD–RhoA interactions.

**Figure 2:**
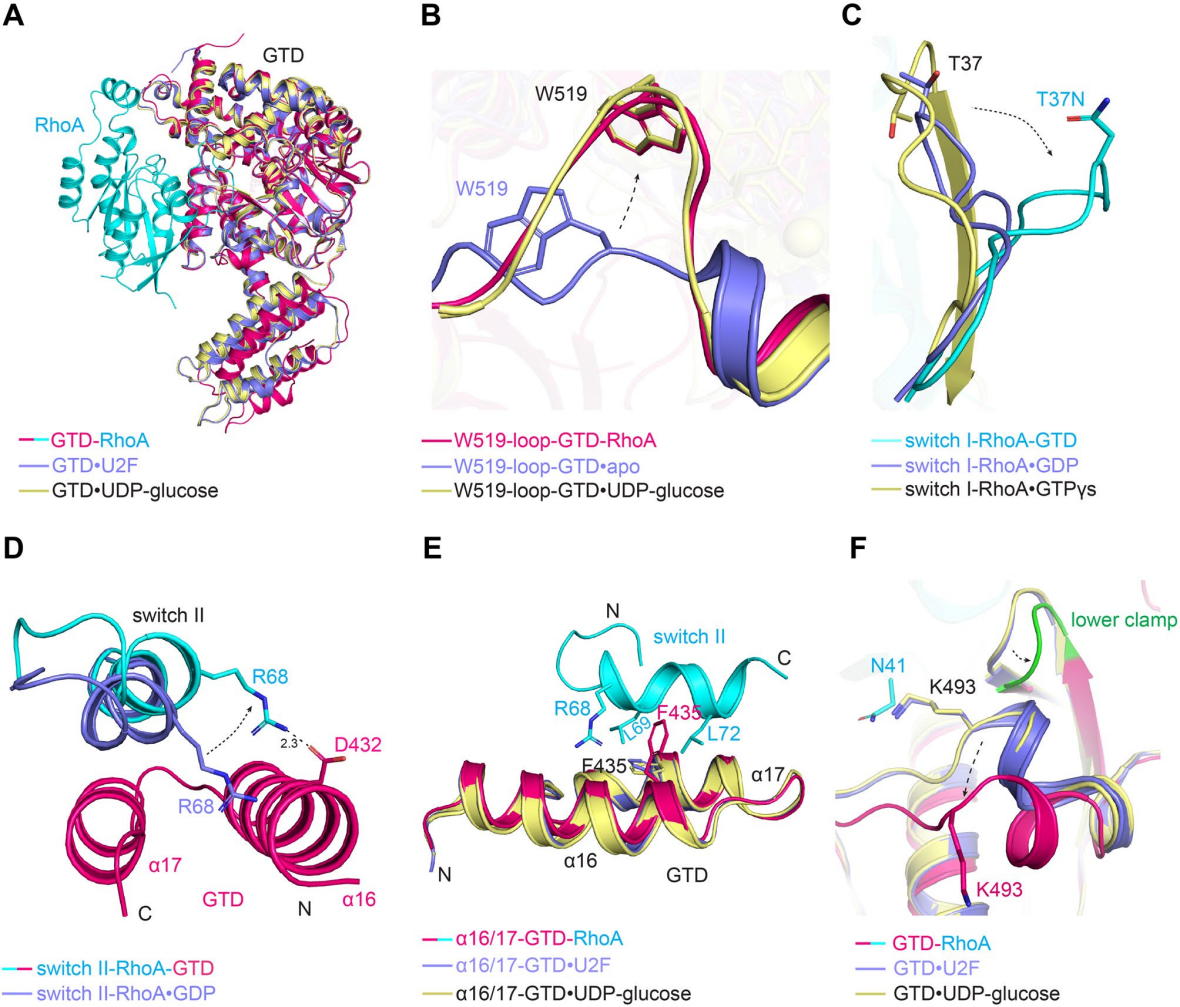
Conformational changes on GTD^TcdA^ and RhoA induced by complex formation. (**A**) Structural superposition of the GTD^TcdA^–RhoA (hot pink and cyan, respectively) complex, GTD^TcdA^·U2F (slate, PDB code: 5UQL), and GTD^TcdA^·UDP-glucose (pale yellow, PDB code: 3SRZ) based on the GTD. (**B**) The W519-loop of GTD^TcdA^ adopts a similar conformation in the GTD^TcdA^·UDP-glucose–RhoA complex (hot pink) and GTD^TcdA^·UDP-glucose (pale yellow), which is drastically different from that of the apo GTD^TcdA^ (slate, PDB code: 4DMV). (**C**) Comparing the conformations of the switch I of RhoA in the GTD^TcdA^–RhoA·GDP complex (cyan), RhoA·GTPγs (pale yellow, PDB code: 1A2B), and RhoA·GDP (slate, PDB code: 1FTN). (**D**) Comparing the conformations of the switch II of RhoA in the GTD^TcdA^–RhoA·GDP complex (cyan) and RhoA·GDP (slate, PDB code: 1FTN). (**E**) Superposition of RhoA-bound GTD^TcdA^·UDP-glucose (hot pink), GTD^TcdA^·U2F (slate) and GTD^TcdA^·UDP-glucose (pale yellow) focusing on the α16/17 helixes. (**F**) GTD^TcdA^ adopts conformational changes to accommodate the pre-switch I and switch I of RhoA when compared to the GTD^TcdA^·U2F (slate) and GTD^TcdA^·UDP-glucose (pale yellow) structures.

The crystal structure shows that GTD^TcdA^ mainly recognizes the switch I (residues 33–42) and switch II (residues 61–75) regions of RhoA (Fig. 1B,C, Fig. S1A,B), and the overall architecture of the GTD^TcdA^–RhoA complex is very similar to the GTD^TcdB^–Cdc42/R-Ras complex (PDB code: 7S0Y, 7S0Z)^20^. The overall structure of RhoA-bound GTD^TcdA^·UDP-glucose is highly similar to the previously reported GTD^TcdA^·UDP-glucose (PDB code: 3SRZ)^25^ and GTD^TcdA^·U2F (a non-hydrolysable UDP-glucose homolog, PDB code: 5UQL)^32^ complexes, with a root mean square deviation (RMSD) of ~ 0.525/0.504 Å over 408/407 residues, respectively (Fig. 2A). Of note, a loop connecting α20 and α21 helices (residues 514–522, referred to as W519-loop) of GTD^TcdA^ adopts a large conformational change upon UDP-glucose·Mn^2+^ binding in comparison to the apo state (PDB code: 4DMV) with the Cα of W519 moving ~ 7 Å (Fig. 2B, Fig. S1C). This movement of the W519-loop is triggered by its direct interactions with UDP-glucose and Mn^2+^^25,32,33^. Upon RhoA binding, the UDP-glucose·Mn^2+^-bound conformation of the W519-loop is further stabilized by residues Y34 and T37N on the switch I of RhoA (Fig. 1D), whereas the apo conformation of the W519-loop would clash with RhoA. The homologous W520-loop on GTD^TcdB^ exploits a similar movement to recognize Cdc42 and R-Ras^20^. These findings suggest that, besides being a glucose donor, UDP-glucose facilitates the GTD of both TcdA and TcdB to engage their substrates.

### Structural basis for RhoA recognition by the GTD of TcdA

We next examine the detailed interactions between GTD^TcdA^ and RhoA, which are mainly mediated by the switch I, switch II, and a region right upstream of the switch I of RhoA (residues 27–32, referred to as pre-switch I) (Fig. S1D). A prominent feature of the GTD-bound RhoA is that its switch I adopts a unique conformation that is different from its GTP-bound active form^34^ or its GDP-bound inactive form^35^, even though it was crystallized in the presence of GDP and Mg^2+^ (Fig. 2C). In the GTD^TcdA^–RhoA complex, the RhoA switch I is stabilized by extensive hydrophobic packing involving residues V33, Y34, V35, P36, V38 and F39 of RhoA and residues M313, A377, L378, V381, I382, I465, P470, A474, L510 and I515 of GTD^TcdA^, suggesting that this new conformation of the switch I is induced by the GTD (Figs. 1D,E, 2C, Table S2). Despite discontinuity in the primary sequence, these GTD^TcdA^ residues converge in 3D to form a largely hydrophobic groove to accommodate the switch I of RhoA, which is further supported by additional hydrogen bonds involving residues Y34, F39, E40 and N41 of RhoA and K448, T491 and E514 of GTD^TcdA^ (Fig. 1D,E, Table S2).

Triggered by the GTD, such a movement of the switch I positions N37 of RhoA, corresponding to the glucosylation target T37 in the wild type RhoA, into the UDP-glucose binding pocket of the GTD, where N37 interacts with residues R462, S517 and S520 of GTD^TcdA^ and UDP-glucose via hydrogen bonds (Fig. 1D). As the engineered RhoA T37N does not have the hydroxyl group to accept the glucosyl unit from UDP-glucose, the structure captured by our crystal structure probably represents a catalysis intermediate state where T37 on wild-type RhoA is primed to be glucosylated.

The second major interface between GTD^TcdA^ and RhoA is between the switch II region of RhoA and the α16/17 helices of GTD^TcdA^ (Fig. 1C). More specifically, residues H431, F435, A438 on the α16 helix of GTD^TcdA^ and residues S443, L446, T447 and A450 on the α17 helix form a hydrophobic pocket to anchor residues L69 and L72 on the switch II of RhoA (Fig. 1F, Fig. S1E). We also observed that residues G62–L69 of the RhoA switch II exhibited a noticeable reorientation to accommodate GTD^TcdA^ binding. For example, R68 of RhoA forms a salt bridge with D432 of GTD^TcdA^, while the conformation of the standalone RhoA would clash with GTD^TcdA^ (Fig. 2D). In contrast, the α16/17 helices of GTD^TcdA^ show an almost identical conformation regardless of RhoA binding except for some subtle sidechain reorientation. For example, the side chain of F435 of GTD^TcdA^ exhibited a movement to better interact with RhoA R68 and L72 and avoid potential clash with L69 (Fig. 2E).

The third interface is established between the pre-switch I of RhoA and two discrete regions in the GTD that form a clamp-like motif, whereas the upper and lower clamps in GTD^TcdA^ are composed of residues 307–310 and 378–380, respectively (Fig. 1B, Fig. S1F,G). Most of the interactions with the pre-switch I are mediated by the lower clamp via hydrogen bonds and hydrophobic packing (Fig. 1E). Furthermore, we observed that both the lower clamp and a nearby loop and helix (residues 489–498) of GTD^TcdA^ reorient upon RhoA binding to better recognize the substrate (Fig. 2F). For example, the bulky side chain of K493 of GTD^TcdA^ moves ~ 7.1 Å to avoid conflicting with N41 of RhoA (Fig. 2F, Fig. S1H).

### Comparison of the Rho-binding modes between TcdA and TcdB

**Figure 3:**
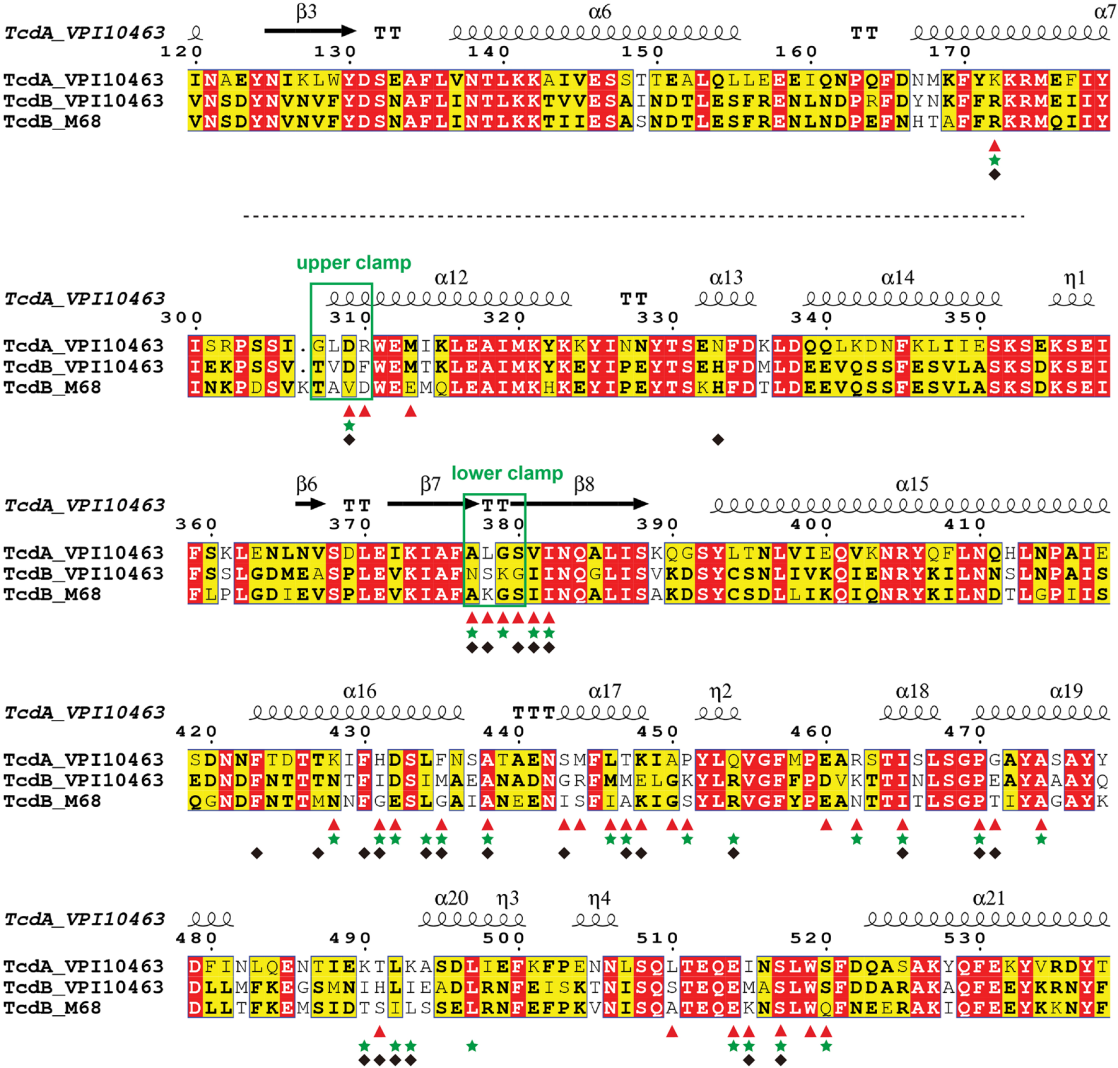
Amino acid sequence alignment among the GTDs of TcdA and two TcdB variants. Residue numbers and the secondary structures of the RhoA-bound GTD^TcdA^ are shown on the top. The residues on the GTDs of TcdA-VPI10463, TcdB-VPI10463, and TcdB-M68 that interact with RhoA, Cdc42, and R-Ras are highlighted with red triangles, green stars, and black rhombuses, respectively. The green boxes highlight the upper and lower clamps of the GTD.

One of the fascinating features of TcdA and TcdB is their abilities to target different GTPases that are key modulators of diverse signaling pathways. For example, variants of TcdB from diverse *C. difficile* strains display different selectivity towards Rho or Ras family GTPases, which are linked to two distinct types of cytopathic effects^36–39^. In our earlier studies using TcdB GTDs from two different strains VPI10463 and M68 as models, we found that GTD^TcdB^ evolves selective clustering of adaptive mutations in the GTPase-binding sites to adjust their specificities toward Rho or R-Ras, while they share a high sequence identity up to ~ 79% among different variants^20^. But since the sequence identity between the GTD of TcdA and TcdB from VPI10463 strain is only ~ 51%, how do they manage to recognize the same set of Rho family members such as RhoA, Rac1, and Cdc42? Now that the structures of the GTD–Rho complexes are available for both TcdA and TcdB, they provide a unique opportunity to investigate both the conserved and divergent features of these two toxins in terms of substrate recognition (Fig. 3, Table S2).

**Figure 4:**
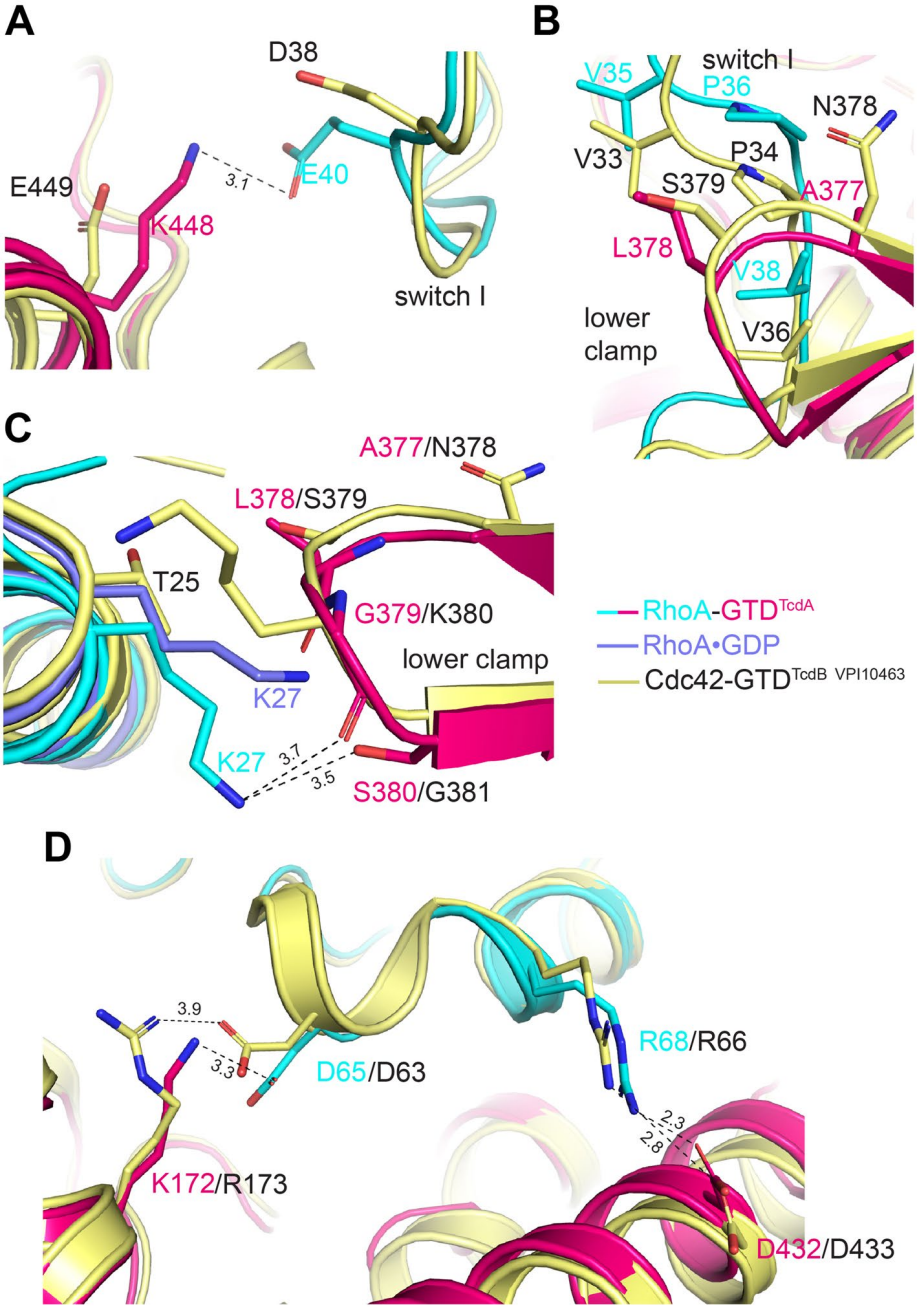
Structural comparison of the GTD^TcdA^–RhoA and the GTD^TcdB-VPI10463^–Cdc42 complexes. (**A**) K448 of GTD^TcdA^ (hot pink) but not the equivalent E449 of GTD^TcdB^ (pale yellow, PDB code: 7S0Y) form a salt bridge with E40 (cyan) of RhoA and potentially D38 of Cdc42 (pale yellow). (**B**) Residues A377 and L378 on the lower clamp of GTD^TcdA^ (hot pink) interact with the switch I of RhoA better than the equivalent N378 and S379 on GTD^TcdB-VPI10463^ (pale yellow, PDB code: 7S0Y). (**C**) Examining the interactions between the pre-switch I of RhoA (cyan) and the lower clamp of GTD^TcdA^ (hot pink) in the GTD^TcdA^–RhoA complex when compared to the GTD^TcdB-VPI10463^–Cdc42 complex (pale yellow) and RhoA·GDP (slate, PDB code: 1FTN). (**D**) Comparing the interactions in the switch II area for the GTD^TcdA^–RhoA (hot pink and cyan, respectively) and the GTD^TcdB-VPI10463^–Cdc42 (pale yellow) complexes.

We first focused on the interfaces where the GTD recognizes the switch I and the pre-switch I areas. We found that most of the Rho-binding residues in this area are conserved between TcdA and TcdB. For example, residues I382, I465, P470, A474, E514, S517, W519, and S520 of GTD^TcdA^ interact with residues Y34, P36, T37N, V38 and F39 on the switch I of RhoA, and all these interacting residues are conserved on GTD^TcdB-VPI10463^ and Cdc42, respectively (Fig. 3, Fig. S3, Table S2). However, there are some interactions unique for the GTD^TcdA^–RhoA complex. For example, K448 of GTD^TcdA^ forms a salt bridge with RhoA E40, which could be applied to interactions with Cdc42 and Rac1 that have a homologous substitution of D38 (Fig. 4A). But GTD^TcdB-VPI10463^ has E449 at the corresponding position that would weaken this engagement. We also noticed that a hydrophobic surface composed of residues I382, I465 and P470 on GTD^TcdA^ that is also conserved on GTD^TcdB^ (I383, I466 and P471) is expanded by residues A377 and L378 on the lower clamp of GTD^TcdA^, which enhance interactions with RhoA residues V35, P36 and V38 on the switch I, as well as residues I23 and P31 on the pre-switch I. In contrast, GTD^TcdB-VPI10463^ has hydrophilic N378 and S379 at the corresponding positions on the lower clamp (Figs. 3, 4B). Moreover, the presence of L378 of GTD^TcdA^ may induce the movement of the bulky side chain of K27 of RhoA on the pre-switch I in order to avoid a clash, and K27 subsequently forms hydrogen bonds with G379 and S380 of GTD^TcdA^ that strengthen interactions (Fig. 4C). This is likely unique to RhoA as Cdc42 and Rac1 have a small Threonine in the place of K27. This finding suggests that the lower clamp of GTD^TcdA^ may match the pre-switch I and switch I of RhoA better than that of GTD^TcdB^, which might partly explain the observation that GTD^TcdA^ was more efficient than GTD^TcdB^ at modifying RhoA based on a time course in vitro experiment^25^.

We then turned our attention to the Rho switch II binding area. Even though the sequences in the switch II are identical for Rho proteins, RhoA·GDP and Cdc42·GDP exhibited slightly different conformations in this area, suggesting some degree of flexibility^35^ (Fig. S2A). Interestingly, the switch II is fixed to an identical conformation upon GTD binding in both cases of TcdA and TcdB (Fig. S2B). We found that most of the interactions in this area are similar on GTD^TcdA^ and GTD^TcdB-VPI10463^. For example, (1) two pairs of salt bridges between K172 of GTD^TcdA^ and RhoA D65, as well as D432 of GTD^TcdA^ and RhoA R68 are identical to that observed in GTD^TcdB-VPI10463^ and Cdc42 (Fig. 4D); (2) P451 of GTD^TcdA^ forms a hydrophobic packing against RhoA Y66, which is corresponding to a cation-pi interaction between K452 of GTD^TcdB-VPI10463^ and Cdc42 Y64; (3) GTD^TcdA^ uses residues H431, A450, F435 and L446 to anchor hydrophobic L69 and L72 on RhoA switch II, which are replaced by homologous residues I432, G451, M436 and M447 on GTD^TcdB-VPI10463^.

## Discussion

This work complements our previous studies of GTPase recognition by GTD^TcdB^^20^, which together provide a more complete understanding of substrate binding mechanism for TcdA and TcdB. Based on structural and sequence analyses, we found that, despite only ~ 51% overall sequence identity across the whole domain, GTD^TcdA^ and GTD^TcdB^ exploit a similar strategy to target Rho proteins. Moreover, the GTD^TcdA^-binding residues on RhoA are largely conserved on Rac1 and Cdc42 (Fig. S3, Table S2), which suggest that GTD^TcdA^ may adopt a similar binding pattern to recognize Rac1 and Cdc42. At the same time, structural comparison between the GTD^TcdA^–RhoA complex and the GTD^TcdB-M68^–R-Ras complex also provides new insights into how GTD^TcdA^ may target H/N/K-Ras as its minor substrates^27,28,40^. Based on structural modeling, we were able to map the potential interacting residues on GTD^TcdA^ and H/N/K-Ras, respectively, which reveals interactions that are conserved between GTD^TcdA^ and GTD^TcdB^ in terms of Ras binding, as well as adaptive residue changes on both GTD^TcdA^ and H/N/K-Ras that may establish unique pair-wise interactions **(**Table S3). This is consistent with our early findings that the GTDs from diverse TcdB natural variants use a common binding mode to target Rho and Ras GTPases by evolving selective amino acid changes at the substrate-binding interface to adjust its substrate specificity^20^. We expect that the comprehensive structural information reported here and in our earlier work will provide a blueprint to guide future mutagenesis and functional studies, which will reveal a more complete understanding of the glucosyltransferase activities and substrate selectivity of TcdA and TcdB and their contributions to *C. difficile* pathogenesis.

## Methods

### Protein expression and purification

The genes encoding RhoA (residues 1–181, Addgene, plasmid #12959) and TcdA GTD (residues 1–542, strain VPI10463) connected by a peptide linker (GGGGSGGGSGTGSGGGGS) were cloned into the pGEX6p-1 vector via *BamH* I/*Xho* I restriction sites. The T37N mutation on RhoA and K190A mutation on TcdA GTD were introduced via QuikChange and verified by DNA sequencing.

The recombinant protein was overexpressed in *E. coli* strain BL21-star (Invitrogen). Bacteria were cultured at 37 °C in LB medium containing ampicillin. Protein expression was induced with 1 mM isopropyl-β-d-thiogalactopyranoside (IPTG) when cell density (OD_600_) reached ~ 0.8. The temperature was then reduced to 18 °C, and the protein expression continued at 18 °C for 18 h. Cells were harvested by centrifugation and stored at − 80 °C for future use.

For purification, cell pellets were re-suspended in a buffer containing 50 mM HEPEs, pH 7.4, 400 mM NaCl and lysed by sonication, and the fusion protein was purified by Glutathione Sepharose resins (Genesee Scientific). The GST-tag was removed by overnight on-column PreScission Protease cleavage at 4 °C and the flow through was collected, which was exchanged into a buffer containing 20 mM Tris, pH 8.5, 40 mM NaCl, and further purified using Mono-Q ion-exchange chromatography (GE Healthcare). The peak fractions were pooled and exchanged into a buffer containing 20 mM Tris, pH 8.0, 150 mM NaCl, 2 mM MgCl_2_, 2 mM MnCl_2_, 0.05 mM GDP, and 2 mM UDP-glucose, which was further concentrated to ~ 10 mg/ml for crystallization.

### Crystallization

Initial crystallization screening of the GTD^TcdA^–RhoA complex was carried out at 18 °C using a Gryphon crystallization robot (Art Robbins Instruments) with sparse matrix screening kits from Hampton Research and Qiagen using the sitting-drop vapor diffusion method (0.2 μl protein + 0.2 μl reservoir equilibrated against 50 μl reservoir). The best crystals were obtained in a condition containing 0.2 M ammonium sulfate, 0.1 M MES, pH 5.9, and 10% (w/v) PEG 8000, after manual optimization and streak-seeding. Crystals were cryoprotected in the mother liquor supplemented with 25% (v/v) ethylene glycerol and snap frozen in liquid nitrogen for data collection.

### Data collection and structure determination

The X-ray diffraction data were collected at 100 K at the NE-CAT beamline 24-ID-C, Advanced Photon Source. The data were processed using XDS as implemented in RAPD (https://github.com/RAPD/RAPD)^41^. The structure was solved by molecular replacement using TcdA GTD (PDB code: 3SRZ)^25^ and RhoA (PDB code: 1FTN)^35^ as search models. One GTD^TcdA^–RhoA complex was positioned in the asymmetric unit using PHENIX.Phaser-MR^42^. All refinement and model building procedures were carried out with PHENIX.refine^43^, refmac5^44^, and COOT^45^. All the refinement progress was monitored with the free R value using a 5% randomly selected test set^46^. The structure was validated by MolProbity^47^. Table S1 shows the detailed statistics of data collection and refinement. All the structure figures were prepared by PyMOL (DeLano Scientific).

## Supplementary Information

Supplementary Information.

## Data Availability

The coordinates and structure factors for the GTD^TcdA^–RhoA complex has been deposited to the Protein Data Bank under access code 7U2P. All other relevant data are within the manuscript and the Supplemental information.
