# Supplementary material for: Structure of the glucosyltransferase domain of TcdA in complex with RhoA provides insights into substrate recognition
Source: Sci Rep. 2022 May 30;12:9028. doi: 10.1038/s41598-022-12909-8 (PMC9151644; doi:10.1038/s41598-022-12909-8)
Supplement: Supplementary file 1 — Supplementary Information. [file 41598_2022_12909_MOESM1_ESM.pdf]

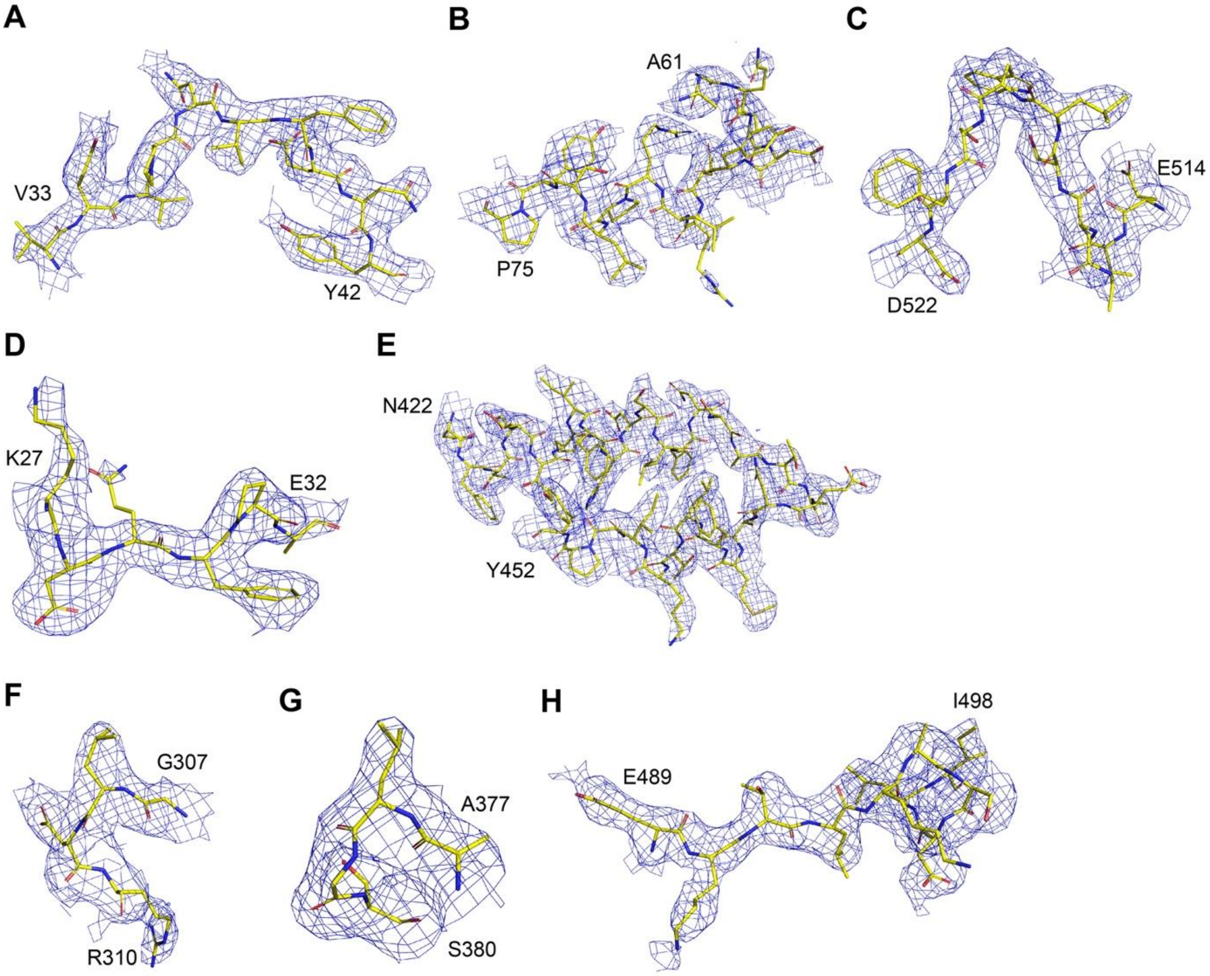

Figure S1 Representative electron density maps of the GTD^{TcdA}-RhoA complex.

The 2Fo-Fc electron density maps showing the switch I (**A**), switch II (**B**), and pre-switch I (**D**) of RhoA; and the W519 loop (**C**), α16/17 (**E**), lower clamp (**F**), upper clamp (**G**), and region 489-498 (**H**) of TcdA GTD. Residues are shown as sticks, density maps are colored blue and contoured at 0.8 σ.

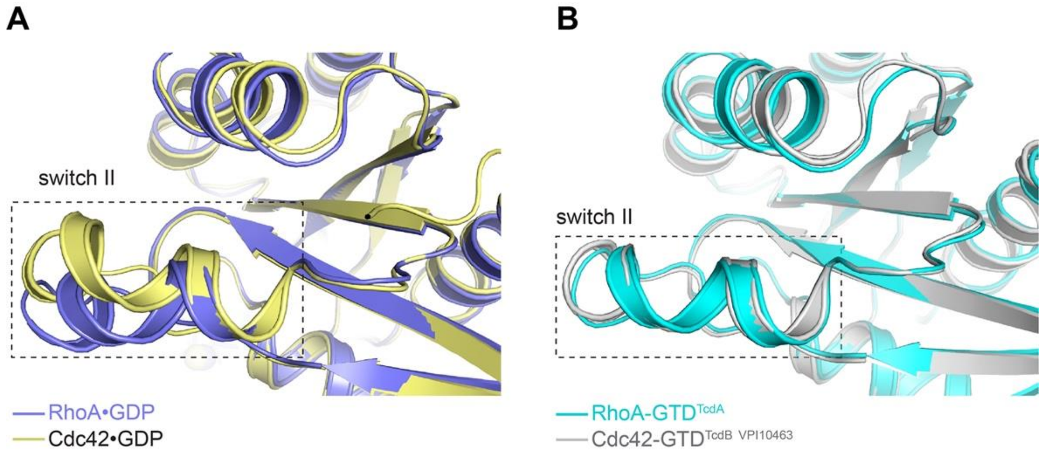

Figure S2 Structural comparison of RhoA and Cdc42 focusing on the switch II.

A. The switch II of RhoA•GDP (slate, PDB code: 1FTN) and Cdc42•GDP (pale yellow, PDB code: 1AN0) adopts slightly different structure.

B. The conformations of the switch II of the GTD^{TcdA}-bound RhoA (cyan) and the GTD^{TcdB}-bound Cdc42 (gray, PDB code: 7S0Y) are virtually identical.

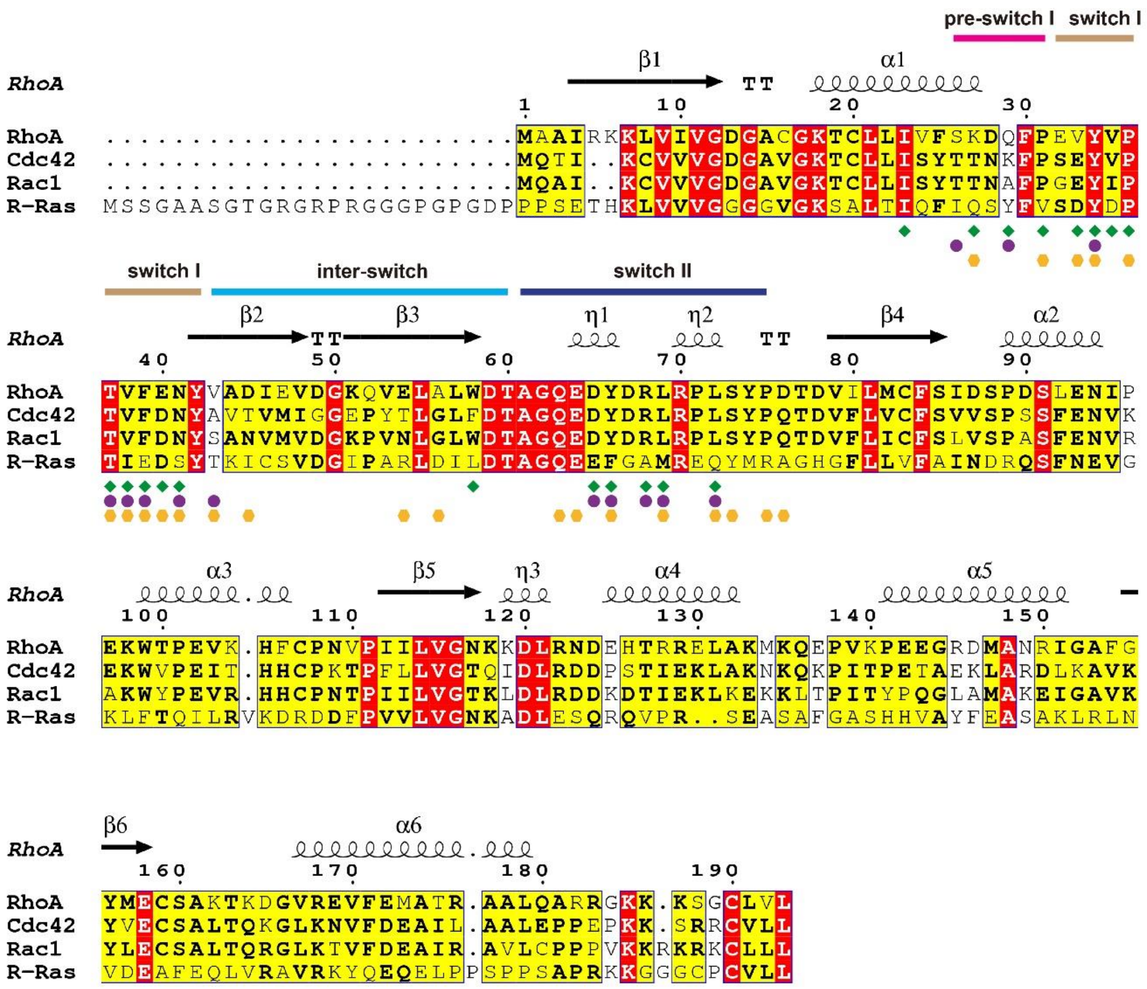

Figure S3. Amino acid sequence alignment among RhoA, Cdc42, Rac1, and R-Ras.

Residue numbers and the secondary structures of the GTD^{TcdA}-bound RhoA are shown on the top. The residues of RhoA, Cdc42, and R-Ras that interact with the GTDs of TcdA, TcdB-VPI10463, and TcdB-M68 are highlighted with blue rhombuses, purple dots, and orange hexagons, respectively.

Table S1. Data collection and refinement statistics.

**Data Collection**	**TcdA GTD-RhoA**
Space Group	C 2 2 21
Cell Dimensions	
a, b, c (Å)	114.64, 122.80, 127.47
α, β, γ (°)	90.00, 90.00, 90.00
Wavelength (Å)	0.9792
Resolution (Å)	63.74-2.60 (2.69-2.60)*
R_{pim}	0.057 (0.525)
Wilson B-factor (Å^2)	57.42
Mean $I/\sigma(I)$	10.79 (1.36)
$CC_{1/2}$	0.997 (0.587)
Completeness (%)	98.5 (97.2)
Redundancy	3.8 (3.5)
**Refinement**	
Resolution (Å)	63.74-2.60 (2.69-2.60)
No. of reflections	27593 (2667)
$R_{\text{work}}/R_{\text{free}}$	0.2149 (0.3303) / 0.2354 (0.3721)
No. of atoms	5933
Protein	5838
Ligand/ion	43
Water	52
B-factor (Å^2)	60.43
Protein	60.46
Ligand/ion	58.57
Water	53.09
Ramachandran plot	
Favored (%)	97.49
Allowed (%)	2.51
Outliers (%)	0.00
R.m.s. deviations	
Bond lengths (Å)	0.008
Bond angles (°)	1.230

* Values in parentheses are for the highest-resolution shell.

Table S2. Protein-protein interactions between GTD^{TcdA} and RhoA$^{\#}$.

TcdB GTD^{M68}	TcdB GTD^{VPI10463}	TcdA GTD^{VPI10463}	Interaction type	RhoA pre-switch I	Cdc42	Rac1
K380	S379	L378	vdW	I23	I21	I21
K380	S379	L378	vdW			
G381	K380	G379 (mc)	HB, 3.7 Å	K27	T25	T25
S382	G381	S380	HB, 3.5 Å			
K380	S379	L378	vdW	Q29	K27	A27
G381	K380	G379 (mc)	HB, 3.7 Å			
K380	S379	L378	vdW	P31	P29	P29
				**switch I**		
V311	D310	D309				
D312	F311	R310	vdW	V33	E31	E31
E315	M314	M313				
A512	S511	L510	vdW			
E516	E515	E514	HB, 3.1 Å	Y34	Y32	Y32
K517	M516	I515	vdW			
K380	S379	L378	vdW	V35	V33	I33
A379	N378	A377				
K380	S379	L378	vdW	P36	P34	P34
I384	I383	I382				
I384	I383	I382	vdW			
N465	K463	R462	HB, 3.1 Å			
I467	I466	I465	vdW			
P472	P471	P470	vdW	T37N	T35	T35
S519	S518	S517	HB, 3.8 Å			
W521	W520	W519	vdW			
Q522	S521	S520	HB, 3.8 Å			
A379	N378	A377				
I383	I382	V381	vdW	V38	V36	V36
I467	I466	I465				
I383	I382	V381 (mc)	HB, 3.0 Å	F39 (mc)	F37	F37
S382	G381	S380				
I383	I382	V381				
I467	I466	I465				
P472	P471	P470	vdW	F39	F37	F37
T473	E472	G471				
A476	A475	A474				
S493	H492	T491				
K450	E449	K448	HB, SB; 3.1 Å	E40	D38	D38
S493	H492	T491	HB, 3.7 Å	N41	N39	N39
				**inter-switch**		
S446	R445	M444	vdW	W58	F56	W56
				**switch II**		
R173	R173	K172	HB, SB; 3.3 Å	D65	D63	D63
E462	D461	E460 (mc)	HB, 3.9 Å	Y66	Y64	Y64
S453	K452	P451	vdW			
N430	N429	K428 (mc)	HB			
G433	I432	H431	vdW	R68	R66	R66
E434	D433	D432	HB, SB; 2.3 Å			
G437	M436	F435	cation-π			
G433	I432	H431				
A449	M448	T447	vdW	L69	L67	L67
G452	G451	A450				
A440	A439	A438				
I445	G444	S443				
G437	M436	F435	vdW	L72	L70	L70
I448	M447	L446				
A449	M448	T447				

$^{\#}$ The corresponding residues on GTD$^{TcdB\text{-}VPI10463}$ and GTD$^{TcdB\text{-}M68}$, as well as Cdc42 and Rac1, are also listed for comparison.

Table S3. Possible protein-protein interactions between GTD^{TcdA} and H/N/K-Ras*.

TcdA GTD$^{\text{VPI10463}}$	TcdB GTD$^{\text{M68}}$	Interaction type	R-Ras pre-switch I	H/N/K-Ras
L378<br>S380	K380 (mc)<br>S382	HB	Q51	Q25
D309	V311	vdW	V55	V29
			**switch I**	
I515<br>I515<br>N333	K517<br>K517<br>H335	HB<br>HB, SB<br>HB, SB	D57 (mc)<br>D57<br>D57	E31
L378<br>WD309	K380<br>V311	HB<br>vdW	Y58 (mc)<br>Y58	Y32
S517	S519	HB	T61N	T35
A377<br>I382<br>I465<br>P470	A379<br>I384<br>I467<br>P472	vdW	P60, I62	P34, I36
V381<br>G471	I383 (mc)<br>T473	HB	E63 (mc)<br>E63	E37
K448<br>K448	K450<br>K450	HB<br>HB, SB	D64 (mc)<br>D64	D38
K490<br>K490	T492 (mc)<br>T492	HB	S65	S39
			**inter-switch**	
L492<br>T491	I494 (mc)<br>S493	HB	T67	R41
K493	L495	vdW	I69	Q43
T491	S493	HB	R78	L52
T491	S493	HB	D80	D54
			**switch II**	
K172	R173	HB	Q87 (mc)	Q61
Q454	R456	HB, SB	E88	E62
F423<br>T427	F425<br>M429	vdW	F90	Y64
F430<br>L434	F432<br>L436	vdW	M93	M67
H431	G433 (mc)	HB	Q96	Q70
T447	A449 (mc)	HB	Y97	Y71
A438<br>F435	A440 (mc)<br>G437 (mc)	HB	R99	R73
S443	I445	vdW	A100	T100

*: Based on structural and sequence comparison with the GTD$^{\text{TcdB-M68}}$-R-Ras complex.

"HB", "SB", "vdW" stand for hydrogen bond, salt bridge, and van der Waals interaction, respectively. "mc" indicates the main-chain mediated contacts, and all the other contacts are mediated by side-chain atoms.
